# Implication of integrin α2β1 in anoikis of SK-Mel-147 human melanoma cells: a non-canonical function of Akt protein kinase

**DOI:** 10.18632/oncotarget.26746

**Published:** 2019-03-05

**Authors:** Nadezhda I. Kozlova, Galina E. Morozevich, Natalia A. Ushakova, Albert E. Berman

**Affiliations:** ^1^ VN Orekhovich Institute of Biomedical Chemistry, Moscow, Russia

**Keywords:** integrins, α2β1, anoikis, tumor growth, Akt isoforms

## Abstract

Suppression of anoikis, a kind of apoptosis caused by disruption of contacts between cell and extracellular matrix, is an important prerequisite for cancer cell metastasis. In this communication, we demonstrate that shRNA-mediated depletion of α2 integrin subunit induces anoikis and substantially decreases colony-forming potential in SK-Mel-147 human melanoma cells. Suppression of α2β1 upregulates the levels of pro-apoptotic protein p53 and cyclin-dependent kinase inhibitors p21 and p27. Concomitantly, we detected decrease in the levels of anti-apoptotic protein Bcl-2 and cell cycle regulator c-Myc. Moreover, depletion of α2β1 reduces the activity of protein kinase Erk, while increases activity of Akt kinase. Pharmacological inhibition of P3IK kinase, an upstream activator of Akt, greatly enhanced anoikis in control cells while reduced that in cells with decreased levels of α2β1. Of three isoforms of Akt, down-regulation of Akt1 greatly diminished anoikis of cells depleted of α2β1, while down-regulation of Akt2 and Akt3 sharply increased anoikis in these cells. These findings were supported by the data of pharmacological inhibition of the Akt isoforms. Our results demonstrate for the first time that anoikis induced by α2β1 integrin knockdown can be attenuated by Akt1 inhibition.

## INTRODUCTION

The growth and malignant progression of tumor cells greatly depends on their interaction with the extracellular matrix (ECM). A hallmark of the malignant phenotype is the capacity of tumor cells to grow in the absence of ECM contacts. Normal cells detached from the ECM, undergo a variant of apoptosis termed anoikis. A critical early phase of tumor progression is the development of mechanisms that enable cells to overcome anoikis [1–3].

Experimental data show that the ECM regulates all aspects of oncogenic transformation. This regulation occurs via a successive trafficking of the matrix-derived signals delivered to nucleus through a chain of intracellular molecules with subsequent modification of gene activity referred to as signal transduction. Key mediators in signal transduction are the integrins, cell surface receptors that directly associate with the matrix proteins and initiate signaling. The majority of studies indicate that integrins are involved in the mechanisms underlying cell behaviors such as proliferation, motility, differentiation, and apoptosis; all of which are modified in the growth and progression of tumors [4–7].

The most common and vital integrins for mammalian cells are fibronectin-binding α5β1, the collagen-binding receptor α2β1 and integrin αvβ3 with diverse ligand specificity. These receptors are the subject of several studies aimed at clarifying the role of integrin-mediated signaling in growth and progression of tumors [8–10].

Only a few reports have addressed the role of integrin α2β1 in anoikis. Along with evidence for the involvement of this receptor in protecting cells from anoikis, there are many reports showing apoptogenic activity of α2β1 in several cell types [11–13]. One possible explanation is that α2β1, like other integrins, can initiate diverse signaling pathways, thereby inducing a variety of responses in different cells depending on factors like their physiological status, developmental stage, interaction with external and internal factors. As signal transduction pathways initiated by integrin α2β1 are not well understood, the present study seeks to demonstrate that integrin α2β1 can be implicated in the protection of tumor cells from anoikis through the inhibition of Akt1, one of the Akt kinase izoforms.

## RESULTS

### Inhibition of integrin α2β1 signaling increased anoikis of SK-Mel-147 melanoma cells and decreased their clonal activity

**Figure 1:**
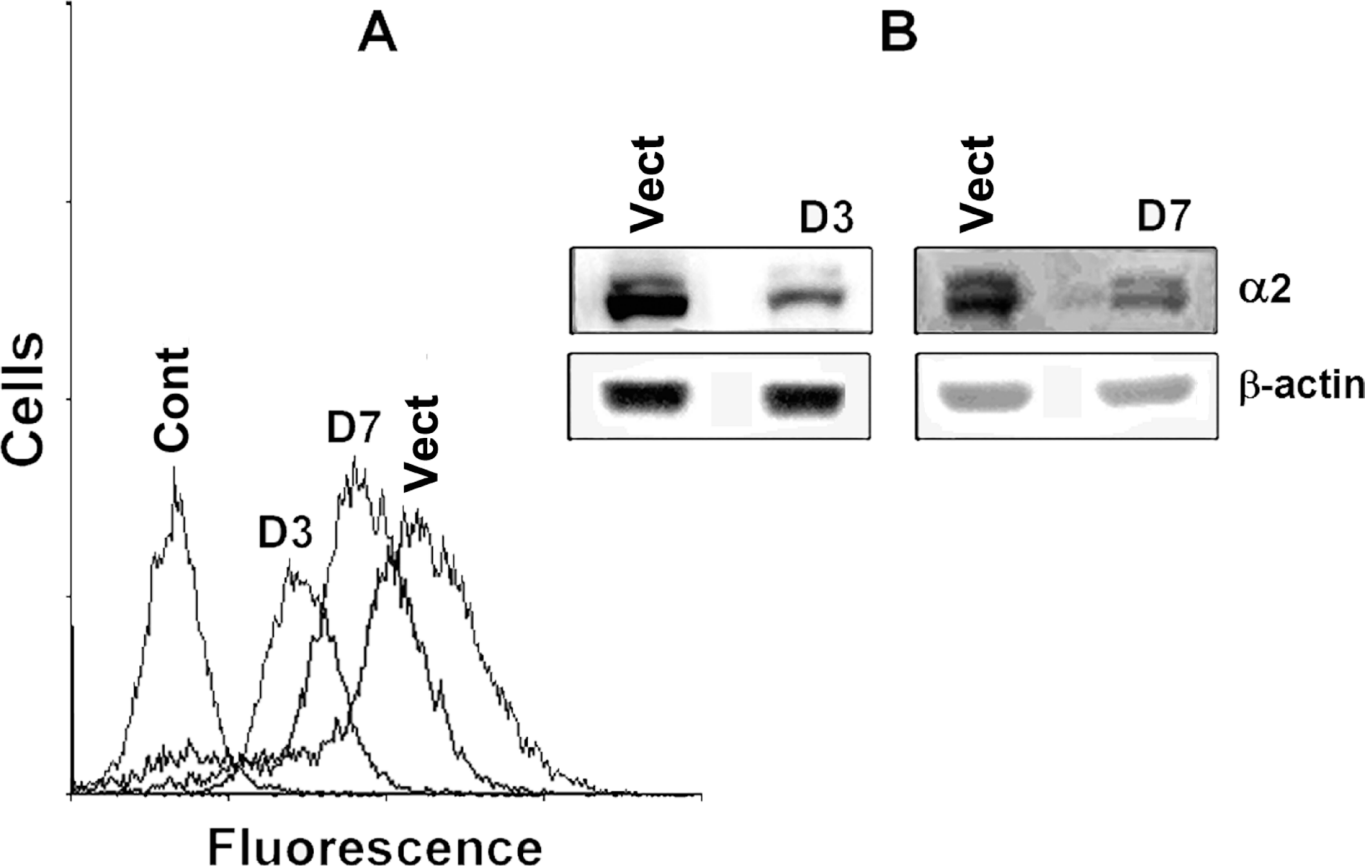
Transduction of SK-Mel-147 cells with α2-specific shRNA effectively inhibits expression of α2β1 integrin The cells were transduced with a lentiviral plasmid vector pLKO.1-puro containing α2 shRNA (clones D3, D7) or with the control vector (the “empty” vector) and selected using puromycin. (A) FACS analysis of α2β1 cell surface expression; Control: cells were transduced with the “empty” pLKO.1-puro and stained with FITC-conjugated anti-mouse IgG; D3, D7: cells were transduced with D3 or D7 clones containing α2 shRNA, treated with α2β1 monoclonal antibodies and stained with FITC-conjugated anti-mouse IgG; Vect: cells were transduced with the “empty” vector, treated and stained as in D3. (**B**) Western-blotting of the cellular lysate proteins. Vect: the cells were transduced with the “empty” vector; D3, D7: the cells were transduced with clones containing α2 shRNA.

To evaluate the implication of α2β1 in cell-matrix detachment-induced stress, we explored the effects exerted on anoikis by down-regulation of this receptor. Signal activity of integrin α2β1 was inhibited by suppressing its expression with the α2-specific shRNA. Investigation of two plasmid clones, expressing α2-specific shRNA, indicated that they both markedly reduced the expression of α2β1 although the D3 clone was more effective (Figure 1).

**Figure 2:**
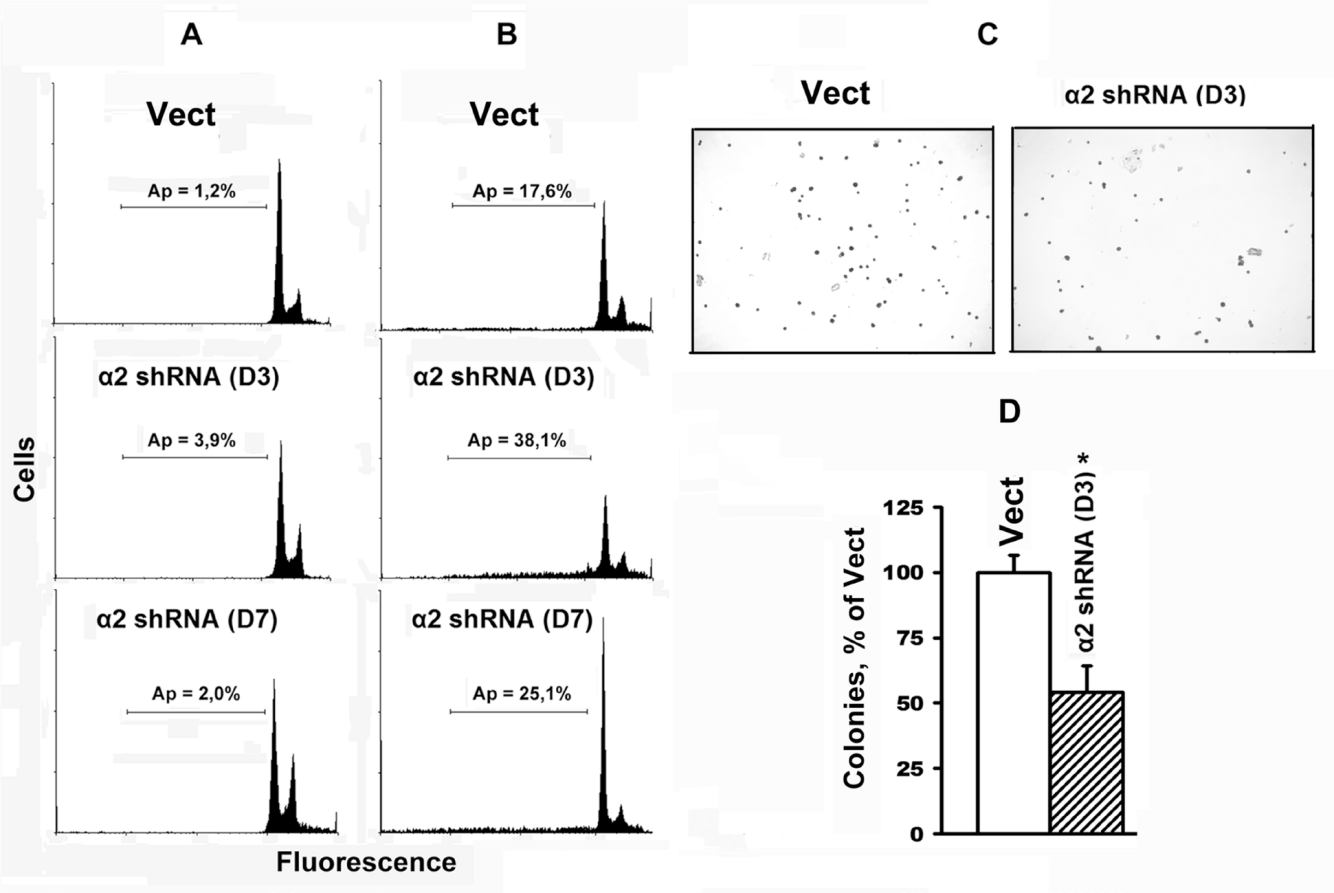
Transduction with α2 shRNA increases anoikis of SK-Mel-147 cells Cells transduced with the empty or α2 shRNA-containing vectors were cultured in a 6-well plate untreated (A) or coated with a non-adhesive substrate – poly-HEMA (**B**) and prepared for flow cytometry. Typical histograms are presented; Ap: apoptotic cells, horizontal lines indicate populations of cells with a sub-diploid (sub-G1) DNA content. Content of apoptotic cells (%) in the total cell population is shown. (**C**, **D**) Effect of α2β1 down-regulation on clonal capacity of SK-Mel-147 cells. Cells tranduced with the empty (Vect) or α2 shRNA-containing vectors (D3, D7), were treated as described in Materials and Methods. C: shown is a typical experiment; D: results of three independent experiments are shown (M ± SEM); *p < 0.01, relative to Vect.

Cells cultured under standard conditions, form and adhere to the substrate consisting of adhesion proteins secreted by these cells (fibronectin, collagen, laminin, etc.). As shown in Figure 2A, SK-Mel-147 cells do not undergo apoptosis irrespectively of their α2β1 level. However, when incubated on a non-adhesive substrate, the cells with lower levels of α2β1 are more prone to anoikis than control cells with higher α2β1; while in control cultures the level of anoikis was relatively small (approximately 17%), in cultures with α2-specific shRNA, the percentage of apoptotic cells increased up to 38% of the total cell population (Figure 2B). Therefore, α2 down-regulation triggers death of SK-Mel-147 cells only on a non-adhesive substrate.

A hallmark of oncogenic transformation of cells is their capacity to form colonies in semi-solid media. A prerequisite for development of this phenotype is resistance to anoikis; however, acquisition of this property depends on the degree of resistance [14]. To characterize the role of α2β1 in oncogenic activity of tumor cells, we analyzed the impact of α2β1 knockdown on the ability of SK-Mel-147 cells to form colonies in methylcellulose gel. The depletion of α2β1 led to a sharp reduction in the number of colonies formed by SK-Mel-147 cells after their cultivation in a methylcellulose gel for 14 days (Figure 2C, 2D). This result corroborates anoikis enhancement in SK-Me-147 cells in response to α2β1 knockdown.

### Signaling pathways that mediate effects caused by inhibition of α2β1

**Figure 3:**
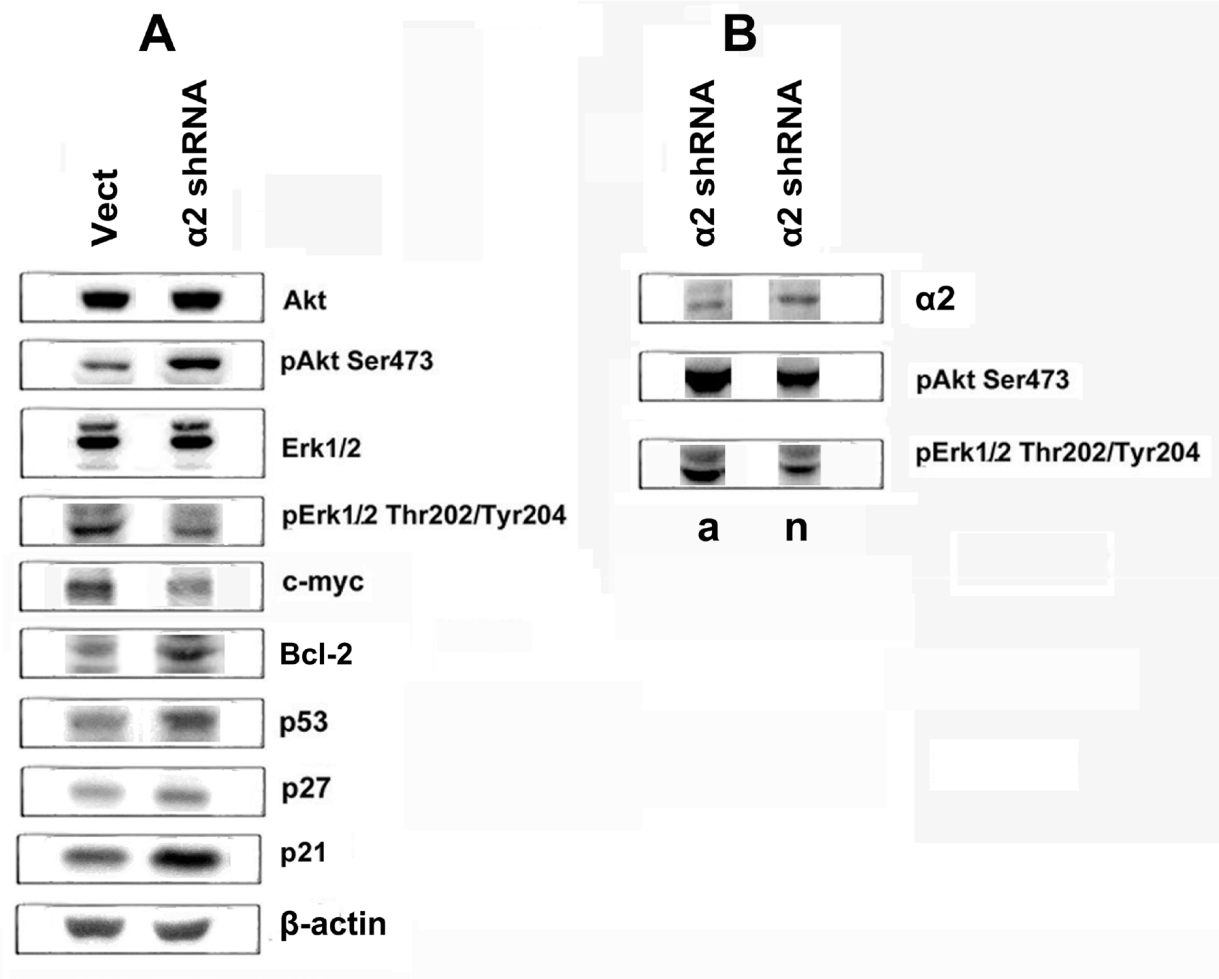
Effect of α2β1 knockdown on expression of signaling proteins in SK-Mel-147 cells (**A**) The cells were transduced with the empty (Vect) or α2 shRNA-containing vectors, and cell lysate proteins were run on SDS-PAGE and western-blotted as described in Materials and Methods. (**B**) α2 shRNA-transduced cells were grown for 24 h on adhesive (a) or non-adhesive (n) substrates and cell lysate proteins were analyzed as in A. The blots were probed with 1:1000 dilution of antibodies to the specified proteins, except for 1:300 dilution of c-Myc antibodies.

To clarify the mechanisms mediating the effect of integrin α2β1 on anoikis, we analyzed the expression of proteins known to be involved in signal transduction and regulation of diverse cellular functions. As shown in Figure 3A, down-regulation of α2β1 leads to a sharp increase in expression of apoptotic p53 and decrease of anti-apoptotic protein BCL-2. In addition, we have found a significant increase in the expression of cell cycle inhibitors, proteins p21 and p27. All of these proteins are known to play important roles in the mechanisms of proliferation and cell survival [15, 16].

These proteins control events which occur in the nucleus, (e.g. the terminal phase of signal transduction) and, therefore, in addition to integrins, other cell receptors and intracellular metabolites can induce these signals. In the case of integrins, more specific are “early” steps of signal transduction which are proximal to cell membranes. Of these pathways, the best characterized are those, mediated by protein kinases IP3-K/Akt and by the MAPK family of kinases, including ERK [17, 18]. To clarify the involvement of these pathways in integrin-mediated signaling, we determined the changes in expression and activity of Akt and ERK1/2 (42- and 44 kDa ERK isomers) kinases which were induced in the α2β1 knockdowned SK-Mel-147 cells. Kinase expression was assessed by western blot of cell lysates using antibodies to the total enzyme protein and their activity was determined with antibodies specific to its active (phosphorylated) forms. As shown in Figure 3A, down-regulation of α2β1 had no effect on the total protein expression of Akt and ERK in melanoma cells, but modified the activity of these kinases. While the cellular amount of phosphorylated ERK isoforms was reduced, the active Akt form substantially increased.

### Non-canonical function of Akt1 in anoikis of SK-Mel-147 cells

The finding of diminished ERK activity in cells with elevated anoikis levels is consistent with the known protective functions of this kinase against the various stresses [18–20]. Since Akt kinase also plays a key role in survival and rescuing the cells under numerous stressful conditions, including anoikis [21, 22], the finding of its activation in cells with a markedly enhanced anoikis might result from the non-canonical Akt functions in these cells, consisting in promoting rather than suppressing anoikis. This assumption agrees with our recently obtained results showing that stimulating effect of α2β1 integrin on the invasion of Sk-Mel-147 cells is mediated through a mechanism based on inhibition of Akt1, one of the isoforms of this protein kinase [23]. But it could not be excluded that the observed increase in Akt activity is a feature accompanying down-regulation of α2β1 in SK-Mel-147 cells and is not related to mechanisms controlling anoikis.

To verify these possibilities, we compared the levels of α2β1 and active Akt in α2 shRNA-treated cells after they had been cultured on adhesive and non-adhesive substrates.

Obviously, the population of cells treated with α2 shRNA is heterogeneous and contains, besides α2 shRNA transduced cells (with reduced level of α2β1 and increased level of pAkt), also a certain number of cells that have not undergone transduction and retained the original levels of α2β1 and pAkt. We hypothesized that if Akt stimulates cell death on a non-adhesive substrate, then cells with lower level of this kinase have a selective advantage and will survive on this substrate. At the same time, some cells with higher Akt levels will die and as such would not contribute to α2β1- and Akt-specific signals in western-blotting. Therefore, one could expect that population of α2 shRNA treated cells, incubated on poly-HEMA, will have higher level of α2β1 and lower level of pAkt than the same population incubated on an adhesive substrate.

As shown in Figure 3B, the population of α2 shRNA-treated cells grown for 24 h on poly-HEMA (as compared with cells grown for the same time on adhesive substrate) demonstrated increased α2β1 and reduced phosopho-Akt levels, while the relative level of active ERK remained unchanged.

**Figure 4:**
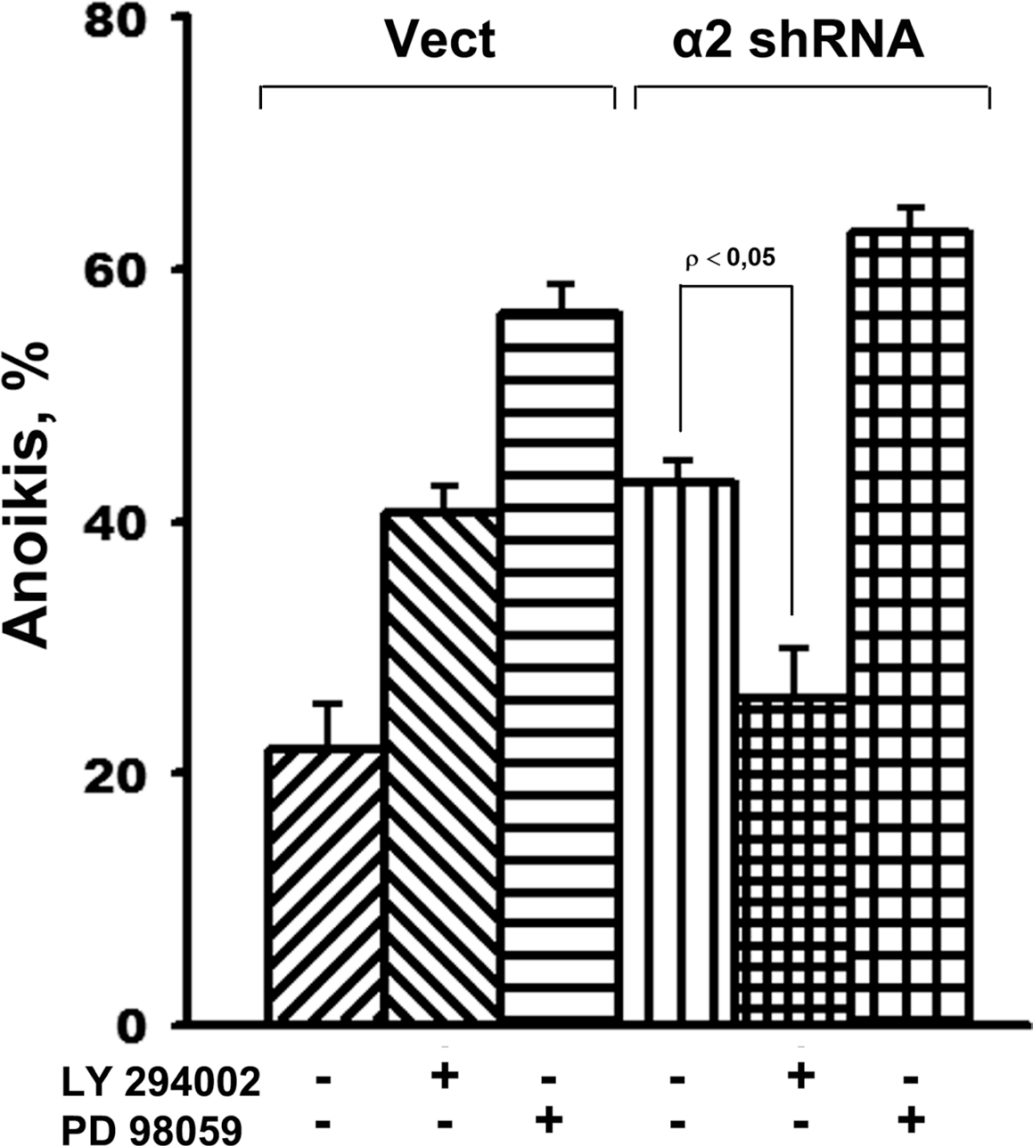
Inhibition of Akt kinase in SK-Mel-147 cells attenuates anoikis, caused by α2β1 knockdown Cells transduced with empty (Vect) or α2 shRNA-containing vectors were cultured on poly-HEMA as described in Materials and Methods. Prior to applying on poly-HEMA the cells were incubated for 24 h at 37° C in the medium supplemented with 25 µM LY294002 or PD98059. Anoikis was determined from percentage of the sub-G1 population. The results of three independent experiments are shown (M ± SEM).

To obtain direct evidence that Akt mediates the death of cells deprived of substrate contacts, the effect of LY 294002, a specific Akt inhibitor, on anoikis was determined in SK-Mel-147 cells with unchanged (control) and reduced α2β1 expression. We supposed that if enhancement of melanoma cell anoikis, induced by α2β1 knockdown, occurs via activation of the PI3K/Akt signal pathway, inhibition of this kinase should neutralize the stimulating effect or even attenuate anoikis. Figure 4 demonstrates that while LY294002 markedly increased anoikis of control cells, it diminished anoikis in α2β1-knockdown cells to a comparable level of anoikis in the control cells. To compare, the Erk inhibitor PD98059 markedly increased anoikis of both the control and α2β1-depleted cells.

In mammals, the AKT kinase family includes three members, AKT1, AKT2 and AKT3, which are encoded by three distinct genes. Recent studies revealed distinct roles of individual AKT members in development of malignant phenotype [24–27]. Notably, LY294002 does not exhibit isozyme-specific effects and suppresses the activity of all isoforms. One could speculate that increased level of phosphorylated Akt found in α2β1-depleted melanoma cells is the result of activation of one of the Akt isoforms, which unlike the other two promotes anoikis, i.e. manifests a non-canonical function.

**Figure 5:**
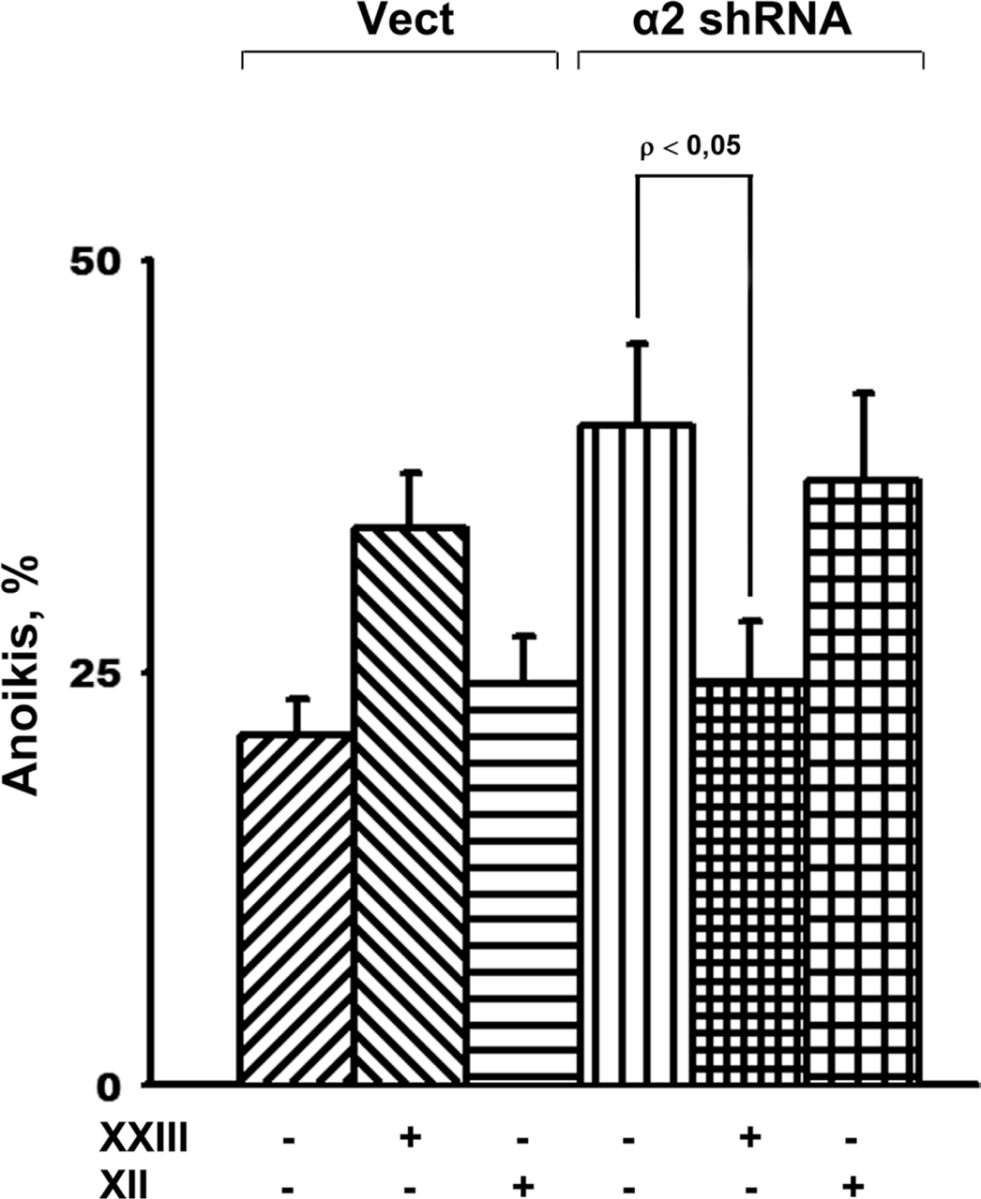
Effect of of Akt isoform inhibitors on anoikis of SK-Mel-147 cells Cells transduced with empty (Vect) or α2 shRNA-containing vectors were cultured on poly-HEMA as described in Materials and Methods. Prior to applying on poly-HEMA the cells were treated for 24 h at 37° C with 3 µM Akt1-specific inhibitor XXIII or 5 µM Akt2-specific inhibitor XII. Anoikis was determined from percentage of Trypan Blue-stained cells. The results of three independent experiments are shown (M ± SEM).

To determine which of the Akt isozymes accounts for the observed effect, we investigated the changes in anoikis in response to inhibition of separate isoforms. As shown in Figure 5, treatment of the cells with compound XXIII resulted in a significant increase of anoikis in the vector-transduced cells and markedly attenuated anoikis in cells transduced with α2 shRNA. Compound XXIII has been previously shown to inhibit all Akt isozymes but possess a 21–23-fold higher potency against Akt1 compared to Akt2 and Akt3, respectively [28]. The concentrations of XXIII used in our experiments were comparable to the IC50 obtained when evaluating the apoptogenic activity of this compound in tumor lines of various origins [28]. Thus, the decrease in anoikis in cells treated with XXIII was most likely due to specific inhibition of Akt1. This suggestion was confirmed by the finding that the Akt2-specific inhibitor, compound XII, unlike XXIII, significantly increased anoikis of the cells transduced with α2 shRNA (Figure 5).

**Figure 6:**
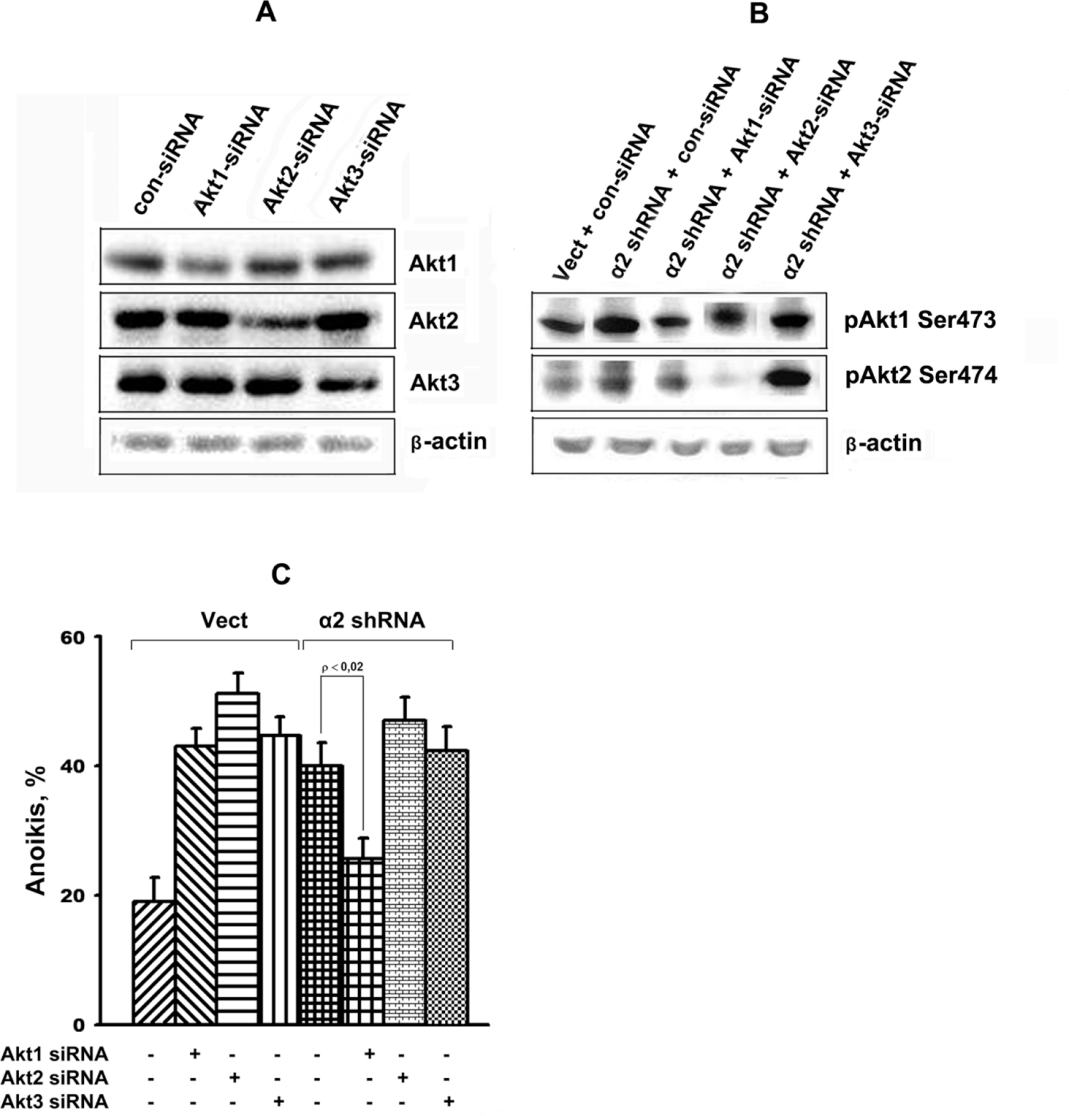
Effect of Akt isoform knockdown on anoikis of SK-Mel-147 cells Cells were transduced with empty (Vect) or α2 shRNA-containing vectors and cultured on poly-HEMA as described in Materials and Methods. Prior to applying on poly-HEMA the cells were transfected with the Akt isoform-specific siRNAs as described earlier [45]. Cell lysate proteins were run on SDS-PAGE, western-blotted and probed with the total Akt isozyme specific antibodies (**A**) or specific to isozyme phosphorylated forms (**B**). Anoikis (**C**) was determined from percentage of Trypan Blue-stained cells. The results of five independent experiments are shown (M ± SEM).

The above studies were supplemented by assessing the changes in anoikis in response to knockdown of each of the three Akt isoforms caused by treating the cells with isoform-specific siRNAs. As shown in Figure 6A, 6B, silencing of either of the Akt isoform effectively down-regulated protein expression and activity of a corresponding isoform. Data in Figure 6C demonstrate that down-regulation of all three Akt isoforms strongly enhanced anoikis of cells transduced with the control vector, while only Akt1 down-regulation lead to a significant decrease in anoikis in the cells transduced with the α2 shRNA.

## DISCUSSION

The present investigation provides the evidence that integrin α2β1 performs a protective function against anchorage-dependent apoptosis (anoikis) in SK-Mel-147 melanoma cells. It was shown for the first time that this receptor triggers a protection mechanism based on inhibition, rather than activation, of Akt kinase. This non-canonical mechanism, not yet described for other integrins, is unexpected because Akt kinase has long been known as a stimulator of proliferation and viability of cells.

Published information on the role of the α2β1 and other integrins in the mechanisms of apoptosis, in particular of anoikis, are scarce and controversial. In different cell models, the same receptor can induce the opposite effects on anoikis. While in acute myeloleukemia cells, cell-surface activation of integrin α4β1 attenuated anoikis [29], its activation in osteosarcoma cells led to anoikis augmenting [30]. A key role of the α5β1 integrin in resistance to anoikis was demonstrated in breast and gastric cancer cells [31]. However, down-regulation, rather than up-regulation, of α5β1 promoted anoikis resistance in a similar model of gastric carcinoma cells [32] and in the cells derived from pancreatic carcinoma [33]. Results of studies on integrin α2β1 are also ambiguous. In our recent publication, down-regulation of the α2β1 integrin lead to a substantial increase of anoikis in human breast carcinoma cells [34]. In a gastric carcinoma line, cell death was shown to be resulted from lectin-induced internalization and inactivation of α2β1 [12]. These findings indicate that the anti-anoikis function of α2β1, are not consistent with the studies on colorectal cancer cells [13]. The authors reported that blocking the expression of histone methyltransferase induced anoikis that was accompanied by a markedly enhanced expression of α2β1, providing indirect evidence for the pro-anoikis capacity of this receptor. The ambiguity of studies on the role of integrins in anoikis is most likely accounted for by their implication in diverse signaling pathways shared with other receptors, which can control opposed cellular responses (proliferation, quiescence, aging, apoptosis, etc.) [35–37].

Only few reports have addressed the detailed characterization of integrin-initiated signal transduction pathways induced upon loss of the cell-matrix contacts. According to Matsunaga, *et al*. [29], resistance of leukemia cells to anoikis is induced by integrin α4β1 through the PI-3K/Akt signal pathway and activation of anti-apoptotic protein BCL-2. Similar properties have been documented for integrin α5β1 in studies on breast carcinoma cells [8]; however, in this study, the evoked signals were transmitted through the MEK-ERK kinases and completed with the suppression of pro-apoptotic protein Bim.

Of considerable interest are the differences found between individual Akt isoforms in their impact on anoikis of melanoma cells. It appeared that of three Akt isoforms only Akt1 demonstrated a non-canonical function which consists in stimulating anoikis of melanoma cells with reduced expression of α2β1 integrin, while two other Akt isoforms had little effect on anoikis of these cells. This result corroborates our recent finding that inhibition of Akt1 (but not Akt2) enhances invasion of the SK-Mel-147 melanoma cells *in vitro* [23].

However, similar non-canonical functions of this kinase have been reported by others in various models of cancer progression. For example, in a breast adenocarcinoma line with high metastatic potential, the effect of TIS21 tumor suppressor, which consists in inhibiting the formation of invadopodia and, therefore, blocking the invasion, is realized through a mechanism mainly based on activation of Akt1, while changes in Akt2 expression had no impact on invadopodia formation and invasive activity [38].

The dual properties of Akt isoforms have been documented in several investigations. Akt1 was shown to stimulate the growth of breast carcinoma but inhibit its metastasis [26]. In contrast, Akt2 enhanced invasion and metastasis of breast and ovarian carcinoma cells [27]. This finding has recently been confirmed on cells of the same origin [25]. It can be assumed that distinct cellular types differ in the expression of individual Akt isoforms, and therefore their responses to Akt-mediated signaling may be different.

Demonstrated in the present communication anoikis-promoting activity of Akt is not a unique property of this signal kinase. We have recently shown that anoikis induced by α2β1 down-regulation in the MCF-7 breast carcinoma cells, is mediated via activation of the ERK kinase [34] and this finding is consistent with a number of studies on various types of cancer cells [39–42]. Thus, integrin-induced signal pathways, evoked in the cells in response to a particular stress impacts are distinct in different neoplasia and need further explorations.

In conclusion, the mechanisms of almost all currently available targeted antitumor drugs are based on suppressing the activity of individual members of the signaling cascades maintaining the transformed phenotypes. Therefore, identification of previously unknown functions of signaling protein kinases is important for cancer therapy. Akt kinase is a central player in the majority of these pathways. For example, Akt inhibitor perifosine demonstrated activity in patients with advanced renal cell carcinoma after failure on VEGF-targeted therapy [43]. As well, perifosin demonstrated efficacy in ovarian cancer and endometrial cancer patients [44]. Obviously, under conditions of pro-death Akt activity, as described by us in the current paper, these agents may cause the opposite effects. Thus, it is important to take into consideration all individual functions of an anticancer target in a specific type of cancer.

## MATERIALS AND METHODS

### Cell culture and chemicals

SK-Mel-147 human melanoma cell line was obtained from the Memorial Sloan Kettering Cancer Center. Cells were cultured in DMEM supplemented with 10% fetal calf serum, 2 mM L-glutamine, penicillin (100 U/ml), and streptomycin (100 μg/ml) and incubated at 37° C in the atmosphere with 5% CO2. Polyclonal antibodies to the α2-integrin subunit and monoclonal antibodies to the integrin α2β1 were, respectively, from Chemicon and BD PharMingen. Polyclonal antibodies to protein kinases Akt, ERK, and their phosphorylated forms (pAkt Ser473, pAkt1 Ser473, pAkt2 Ser474, and pERK Thr202/Tyr204) were from Cell Signaling Biotech. Akt isoform specific siRNAs were from Santa Cruz Biotech. PI3K inhibitor, LY294002, Akt1 inhibitor XXIII, Akt2 inhibitor XII, and ERK inhibitor, PD98059, were from Calbiochem, and other chemicals were from Sigma.

### Transduction of cells with shRNA

Bacterial glycerol clones NM_002203.2-1427s1c1 (#D3) and NM_002203.3-1561s21c1 (#D7) containing lentiviral plasmid vector pLKO.1-puro with shRNA specific for the α2- integrin subunit and pLKO.1-puro lentiviral vector without shRNA («empty» vector, control) were purchased from Sigma. Lentivirus was produced in HEK293T cells by co-transfection with shRNA-containing or control vector with packing plasmids as described earlier [44, 45]. Cells were transduced with lentivirus in the presence of polybrene (8 μg/ml) and selected with puromycin (1–2 μg/ml) for 4–6 days.

### siRNA transfection

siRNA duplexes, both control and specific to the Akt isoforms, as well as siRNA transfection reagent were from Santa Cruz Biotech. (CA, USA). The procedure was performed as described earlier [46]. Briefly, 1–2 × 105 cells in 1 ml of antibiotic-free DMEM + 10% FBS were plated into each well of 6-well culture plates. After reaching ∼50% confluence the cells were transfected with 50 nM siRNA (final concentration) for 70 h. Then cells were harvested with trypsin/EDTA, washed and used for subsequent experiments.

### Anchorage-dependent apoptosis (anoikis)

Anoikis was assessed by the accumulation of cells with DNA content lower than that of diploid cells (sub-G1 population), after their cultivation on the non-adhesive substrate - polyhydroxyethylmethacrylate (poly-HEMA). The substrate was prepared in 6-well plates according to previously described procedure [34]. 3 × 105 cells/well were incubated in medium containing 10% fetal serum, at 37° C for 24 h and prepared for FACS analysis. In some experiments, anoikis was assessed by percentage of cells stained *in vivo* with Trypan Blue, which was determined in an automatic cell analyzer Vi-Cell (Beckman Coulter). Anoikis in these experiments did not differ from that detected by FACS analysis

### Clonal activity

2000 cells were plated on a 1% methylcellulose gel in complete medium in Petri dishes for 14 days. The colonies were stained with Crystal Violet, visualized in an optical microscope and photographed.

### Flow cytometry

1 × 105 cells were fixed with 70% ethanol, rinsed in PBS, resuspended in 1 ml citrate buffer containing 50 μg/ml propidium iodide and 10 μg/ml RNAse A, followed by storing on ice for 3 h. Cell surface expression of α2β1 integrin was assessed by treating the cells with anti-α2β1 (BD PharMingen), followed by staining with FITC-conjugated secondary antibody and fixation with 2% formaldehyde. The cells were analyzed using Becton Dickinson flow cytometer.

### SDS-PAGE and Western-blotting

The procedures were performed as described in [34]. 30 μg of cell lysate proteins were run on SDS-PAGE and electroblotted onto a PVDF membrane. After reaction with specific primary antibodies, the membrane was incubated with HRP-conjugated secondary antibodies, developed in a ECL detection system (Amersham, England) and imaged on MF-ChemiBis 3.2 (DNR Bio-Imaging Systems).

### Statistical analysis

Differences between the groups were assessed using Student’s *t-*test and considered significant at *p* < 0.05.
